# Supplementary material for: Description of global innovative methods in developing the WHO Community Engagement Package
Source: BMJ Open. 2022 Jun 7;12(6):e063144. doi: 10.1136/bmjopen-2022-063144 (PMC9174797; doi:10.1136/bmjopen-2022-063144)
Supplement: Author's manuscript [file bmjopen-2022-063144.draft_revisions.pdf]

BMJ Open is committed to open peer review. As part of this commitment we make the peer review history of every article we publish publicly available.

When an article is published we post the peer reviewers' comments and the authors' responses online. We also post the versions of the paper that were used during peer review. These are the versions that the peer review comments apply to.

The versions of the paper that follow are the versions that were submitted during the peer review process. They are not the versions of record or the final published versions. They should not be cited or distributed as the published version of this manuscript.

BMJ Open is an open access journal and the full, final, typeset and author-corrected version of record of the manuscript is available on our site with no access controls, subscription charges or pay-per-view fees (http://bmjopen.bmj.com).

If you have any questions on BMJ Open's open peer review process please email info.bmjopen@bmj.com

BMJ Open

A Description of Global Innovative Methods in Developing the WHO Community Engagement Package

Journal:	*BMJ Open*
Manuscript ID	bmjopen-2022-063144
Article Type:	Original research
Date Submitted by the Author:	24-Mar-2022

Complete List of Authors:	Bayugo, Yolanda; World Health Organization, Country Readiness Strengthening<br>Labarda, Meredith; University of the Philippines Manila Department of Clinical Epidemiology; University of the Philippines Manila School of Health Sciences<br>Cruz, Jose Rene Bagani; University of the Philippines Manila<br>Mier-Alpano, Jana Deborah; University of the Philippines Manila<br>Tiangco, Pauline Marie; University of the Philippines Manila<br>Oyene, Ukam; World Health Organization<br>Omoleke, Semeeh; World Health Organization, Field Presence; World Health Organization, Country Readiness Strengthening<br>Ulitin, Allan; University of the Philippines Manila,<br>Ong Jr., Alberto; Alliance for Improving Health Outcomes<br>Fajardo, Marvinson; University of the Philippines Manila<br>Echavarria, Maria Isabel; Centro Internacional de Entrenamiento e investigaciones médicas (CIDEIM); Universidad Icesi<br>Alger, Jackeline; Hospital Escuela, ; Instituto de Enfermedades Infecciosas y Parasitologia Antonio Vidal, NA<br>Mathanga, Don; University of Malawi College of Medicine<br>Msiska, Barwani ; Kamuzu University of Health Sciences,<br>Ekwunife, Obinna Ikechukwu; Nnamdi Azikiwe University, Clinical Pharmacy and Pharmacy Management; Nnamdi Azikiwe University, Department of Clinical Pharmacy and Pharmacy Management<br>Nwaorgu, Obioma; Nnamdi Azikiwe University; Nnamdi Azikiwe University, Department of Parasitology and Entomology<br>Abella Lizcano, Lorena; Centro Internacional de Entrenamiento e investigaciones médicas (CIDEIM); Universidad Icesi<br>gomez quenguan, Natalia; Centro Internacional de Entrenamiento e investigaciones médicas (CIDEIM); Universidad Icesi<br>Nieto Anderson, Claudia; SIHI Honduras Hub,<br>Beltran, Briana; Centro de educación medica continua Honduras,<br>Carcamo Rodriguez, Elsy; Independent Researcher, Francisco Morazán; Independent Researcher, Francisco Morazán<br>Núñez, Eduardo; Universidad Católica de Honduras Nuestra Señora Reina de la Paz Facultad de Ciencias de la Salud, Facultad de Ciencias Médicas; Hospital General Santa Teresa, Cirugía General<br>Nkosi-Kholimeliwa, Vera; Evangelical Lutheran Development Services<br>Mwafulirwa-Kabaghe, Glory; Joyful Motherhood, Maternal and Newborn care<br>Juban, Noel; University of the Philippines Manila Department of Clinical Epidemiology

Keywords:	PUBLIC HEALTH, SOCIAL MEDICINE, PRIMARY CARE

SCHOLARONE™
Manuscripts

I, the Submitting Author has the right to grant and does grant on behalf of all authors of the Work (as defined in the below author licence), an exclusive licence and/or a non-exclusive licence for contributions from authors who are: i) UK Crown employees; ii) where BMJ has agreed a CC-BY licence shall apply, and/or iii) in accordance with the terms applicable for US Federal Government officers or employees acting as part of their official duties; on a worldwide, perpetual, irrevocable, royalty-free basis to BMJ Publishing Group Ltd ("BMJ") its licensees and where the relevant Journal is co-owned by BMJ to the co-owners of the Journal, to publish the Work in this journal and any other BMJ products and to exploit all rights, as set out in our licence.

The Submitting Author accepts and understands that any supply made under these terms is made by BMJ to the Submitting Author unless you are acting as an employee on behalf of your employer or a postgraduate student of an affiliated institution which is paying any applicable article publishing charge ("APC") for Open Access articles. Where the Submitting Author wishes to make the Work available on an Open Access basis (and intends to pay the relevant APC), the terms of reuse of such Open Access shall be governed by a Creative Commons licence – details of these licences and which Creative Commons licence will apply to this Work are set out in our licence referred to above.

Other than as permitted in any relevant BMJ Author's Self Archiving Policies, I confirm this Work has not been accepted for publication elsewhere, is not being considered for publication elsewhere and does not duplicate material already published. I confirm all authors consent to publication of this Work and authorise the granting of this licence.

For peer review only

A Description of Global Innovative Methods in Developing the WHO Community Engagement Package

Yolanda Bayugo[1], Meredith del Pilar-Labarda[2,3], Jose Rene Bagani Cruz[3], Jana Deborah Mier-Alpaño[3], Pauline Marie P. Tiangco[3], Ukam Ebe Oyene[1], Semeeh Akinwale Omoleke[1], Allan R. Ulitin[3], Alberto Ong Jr[3], Marvinson Fajardo[3], María Isabel Echavarría[4], Jackeline Alger[5], Don Mathanga[6], Barwani Khaura Msiska[6], Obinna I. Ekwunife[7], Obioma Nwaorgu[7], Lorena Abella[4], Natalia Gómez[4], Claudia Nieto-Anderson[8], Briana Beltran[9], Elsy Cárcamo[10], Eduardo Núñez[10], Vera Nkosi-Kholimeliwa[11], Glory Mwafulirwa-Kabaghe[12], and Noel R. Juban[3,13]

[1] Country Readiness Strengthening Department, Health Emergencies Programme, World Health Organization, Geneva, Switzerland

[2] Department of Medicine, School of Health Sciences, University of Philippines Manila, Philippines

[3] Social Innovation in Health Initiative Philippines, University of the Philippines Manila, Philippines

[4] Centro Internacional de Entrenamiento e Investigaciones Médicas (CIDEIM), Colombia

[5] Facultad de Ciencias Médicas, Universidad Nacional Autónoma de Honduras (UNAH), Tegucigalpa, Honduras

[6] Social Innovation in Health Initiative Malawi, Kamuzu University of Health Sciences, Blantyre, Malawi

[7] Social Innovation in Health Initiative Nigeria, Nnamdi Azikiwe University, Awka, Nigeria

[8] Dirección de Investigación Científica, Universidad Nacional Autónoma de Honduras (UNAH), Tegucigalpa, Honduras

[9] Centro Nacional de Educación Médica Continua, Colegio Médico de Honduras, Tegucigalpa, Honduras

[10] Universidad Nacional Autónoma de Honduras (UNAH), Tegucigalpa, Honduras

[11] Evangelical Lutheran Development Services (ELDS), Malawi

[12] Joyful Motherhood, Lilongwe, Malawi

[13] Department of Clinical Epidemiology, College of Medicine, University of the Philippines Manila, Philippines

Corresponding author: Meredith del Pilar-Labarda, mdlabarda@up.edu.ph

Word count: 3,285

For peer review only

For peer review only - http://bmjopen.bmj.com/site/about/guidelines.xhtml

ABSTRACT

Objectives Development of a Community Engagement Package (CEP) composed of (1) database of community engagement (CE) experiences from different contexts, (2) CE learning package of lessons and tools presented as online modules, and (3) CE workshop package for identifying CE experiences to enrich the CE database and ensure regular update of learning resources. The package aims to guide practitioners to promote local action and enhance skills for CE.

Setting and Participants The packages were co-created with diverse teams from WHO, SIHI, UNICEF, community practitioners, and other partners providing synergistic contributions and bridging existing silos.

Methods The design process of the package was anchored on CE principles. Literature search was performed using standardized search terms through global and regional databases. Interviews with CE practitioners were also conducted.

Results A total of 356 cases were found to fit the inclusion criteria and proceeded to data extraction and thematic analysis. Themes were organized according to rationale, key points and insights, facilitators of CE, and barriers to CE. Principles and standards of CE in various contexts served as a foundation for the CE learning package. The package comprises four modules organized by major themes such as mobilizing communities, strengthening health systems, CE in health emergencies, and CE as a driver for health equity.

Conclusion After pilot implementation, tools and resources were made available for training and continuous collection of novel CE lessons and experiences from diverse socio-geographical contexts.

For peer review only - http://bmjopen.bmj.com/site/about/guidelines.xhtml

STRENGTHS AND LIMITATIONS OF THIS STUDY

- The WHO Community Engagement Package (CEP) was co-created with a community of diverse teams of WHO, Social Innovation in Health Initiative hubs, UNICEF, partners organizations, and community practitioners that provided synergistic contributions in promoting best community engagement (CE) practices across the board.
- This project fills a need for a harmonized CE documentation package for training based on different local contexts and with a broad range of health and social development activities including health emergencies, routine immunization, neglected tropical diseases, city and urban development, nutritional interventions, and disaster risk management.
- The CE cases identified were limited to those in English, French, and Spanish. Future researches can explore relevant documented and undocumented experiences in other languages.
- The CEP was developed and tested primarily through online environments and might need adjustment for in-person implementation.

INTRODUCTION

There is an increasing necessity to redouble efforts using innovative approaches to bolster community engagement (CE) in the global health setting. Emergencies, including the COVID-19 pandemic, severely disrupted prevention and treatment services for non-communicable diseases (NCDs), malaria and other interventions.[1-4] This has compounded health inequities and widened the gap across populations. The complexities brought about by these health problems make community participation in co-creating innovative solutions to these challenges even more critical. The shift to people-centred approaches as highlighted in the revised WHO risk communication and community engagement (RCCE) strategy,[5, 6] is imperative as CE can make a considerable difference in health outcomes and capacitate communities to deal with

For peer review only - http://bmjopen.bmj.com/site/about/guidelines.xhtml

health challenges and their determinants.[7-9] The response to the Nepal earthquake and similar experiences made clear that people-centred design and leadership in addressing problems facilitate more efficient use of resources, strengthen coordination and build local capacities.[10] The World Health Organization (WHO), United Nations Children's Fund (UNICEF) and development partners support CE with resource mobilization, information, and trainings with various outcomes and competencies.[11] However, there is no harmonized CE documentation package based on local contexts for training. This project was initiated to guide health practitioners in promoting local action, and to facilitate involvement, training, and synergies across health and development sectors to achieve collective outputs and outcomes.[12-15] It responds to the need to invest in effective social innovations grounded on CE, which utilize bottom-up approaches and draw on strengths of individuals, communities, and institutions, while promoting synergies across sectors.[16-18]

The WHO Community Engagement Package

The WHO Department of Country Readiness Strengthening conceptualized and initiated the Community Engagement Package (CEP) project based on consultations within WHO Regional Offices and Headquarters. The CEP project[19] developed a database of CE experiences, a CE learning package (CELP), and a CE workshop package (CEWP) based on a broad scope of CE experiences in different settings. The compiled cases can guide program managers, CE practitioners, in-service medical and non-medical trainees, non-governmental organizations (NGO) staff, and multidisciplinary teams to sharpen their skills in the CE approach.

For peer review only - http://bmjopen.bmj.com/site/about/guidelines.xhtml

CEP Project Design and Components

The design of the CEP involved the creation of a database of relevant CE cases. These cases were categorized and analyzed, and themes and concepts were used to develop the CELP with contributions from CE subject matter experts (SMEs). The CEWP was designed to document "newer" CE experiences that can be incorporated into the database, ensuring regular updates of the learning resources (see Figure 1). Table 1 summarizes the three components of the CEP.

Figure 1. WHO Community Engagement Package Components and Relationships

Table 1 ***Descriptions of the Components of the WHO Community Engagement Package***

Community Engagement Database	Organized collection of data and documentation of community engagement experiences, practices, and approaches in different regions and contexts.
Community Engagement Learning Package	Curation of community engagement lessons and tools presented as online (asynchronous) modules designed to capacitate learners on basic concepts, principles, and applications of community engagement, and explore best practice experiences in solving health problems and promoting health through community engagement.
Community Engagement Workshop Package	Provides tools and templates for identifying community engagement experiences in a workshop format. The contents are similar to the Community Engagement Learning Package, with a special focus on documenting "new" CE experiences and their nuances, and a walk-through of using and submitting case studies for the CE database.

Given the uniqueness, relevance, and value of the harmonized CEP in the context of health emergencies and the overall global health sphere, this paper seeks to document

For peer review only - http://bmjopen.bmj.com/site/about/guidelines.xhtml

the processes and the innovative ways by which the CEP was developed at the height of COVID-19 restrictions.

METHODS

Patient and Public Involvement

The conceptualization, design, and conduct of the CEP involved participation and co-creation among colleagues and potential end users in the WHO, SIHI hubs, UNICEF and other implementing partners, and community practitioners and frontline responders.

CEP Human Resource Infrastructure and Way of Working

The overall project methodology was anchored on CE principles and processes. Colleagues in WHO (Headquarters and regions) participated in the CEP project. The Social Innovation in Health Initiative (SIHI) global network contributed substantially to the realization of the CEP.

WHO CEP Working Group

The design of the CEP project came about after consultations with WHO colleagues involved in CE work, bringing in experiences of WHO working with communities in different contexts and settings.[19] These colleagues work in different thematic areas: health promotion, social determinants of health, health systems, disaster risk reduction, risk communication, healthy cities, community readiness and resilience, and population-based focused work. As the CEP design was drafted, a working group (WG) was established to provide technical advice and CE resources related to their respective

For peer review only - http://bmjopen.bmj.com/site/about/guidelines.xhtml

areas of work. Regular WG meetings were conducted to ensure that they had updated information and an opportunity to provide feedback to improve the package. Some members of the WG also participated as resource persons in the CELP.

The WG also consulted and regularly updated the RCCE Collective Services, which is composed of WHO, UNICEF, International Federation of Red Cross and Red Crescent Societies (IFRC) and Global Outbreak Alert and Response Network (GOARN). UNICEF provided inputs regarding training.

SIHI Global Network

The SIHI Philippines Hub is the main implementing agency of the project. It is part of the SIHI global network of research hubs and other partners supported by TDR, the Special Programme for Research and Training in Tropical Diseases. SIHI hubs have expertise and experience documenting social innovations from communities and communicating these innovations with stakeholders.

Led by the SIHI Philippines, the SIHI hubs based in Colombia, Honduras, Malawi, Nigeria, and South Africa also participated in this project. Together, they gathered published and grey literature on CE and were involved in the development of the search terms and selection criteria, case abstracts and identification of themes. SIHI Philippines spearheaded the development of the prototype learning and workshop packages and facilitated regular virtual meetings with the other hubs and WHO staff for updates and consultation.

For peer review only - http://bmjopen.bmj.com/site/about/guidelines.xhtml

Development of the Components of the CEP

The development of the components of the CEP can be characterized as iterative, collaborative and comprehensive and can be considered "community engagement in practice."

Development of the Community Engagement Database

The CE database is an organized collection of data and documentation of CE practices, experiences, and approaches used in different regions and contexts. Systematic search was done to gather and organize these, integrating multi-stakeholder and consultative approaches across the SIHI global network and key partners from WHO.

Search for Materials on Community Engagement

This phase identified materials that document experiences about CE in programs that address health or the social determinants of health. The search procedures were developed and co-created with SIHI hubs and the WHO using the "system lens" principles and a bottom-up approach. Methods were refined as feedback was collected during implementation.

A standard procedure was prescribed for literature search to ensure the quality of cases found and maximize use of search platforms. For published literature (i.e. case reports/series, review articles, research papers, journal articles), searches in PubMed, Google Scholar, Hinari, Research Gate, Scopus, Embase, LILACS were conducted. Other significant local and regional repositories were also explored.

The following standard search terms were used:

For peer review only - http://bmjopen.bmj.com/site/about/guidelines.xhtml

- ("social" OR "community" OR "stakeholder") AND ("engagement" OR "partnerships" OR "participation" OR "action" OR "involvement" OR "empowerment")
- ("social" OR "community") AND ("ownership" OR "relations" OR "outreach")
- "community-based participatory research"
- "population health management"
- "community-directed intervention"

These terms were also translated to French and Spanish and additional terms for a geographic location were also added to focus searches in these areas.

For grey literature (i.e. newsletters, unpublished reports or limited distribution, theses, conference papers/presentations, books, and others) general search engines were used and academic and professional networks were tapped. Materials in languages other than English were included, with interpretation assistance from the SIHI network. Audiovisual materials were collected from credible organizational partners of WHO and SIHI, sources recommended by these organizations, and verified social media accounts and websites.

Interviews, surveys, and correspondence with CE practitioners were facilitated to identify undocumented CE practices. Academic and professional networks of the SIHI network, WHO, and partners were engaged in identifying undocumented CE practices for inclusion. Virtual communication technologies were utilized because of travel restrictions. Recordings or transcripts were obtained for documentation. The reviews were conducted by the project staff and SIHI hubs in coordination with the WG.

For peer review only - http://bmjopen.bmj.com/site/about/guidelines.xhtml

Following PRISMA's recommended process flow, materials collected were screened initially through the title and abstract, when available. These were then assessed based on the selection criteria.

Selection Criteria

A set of criteria (Table 2) was developed to standardize relevant CE cases that were entered into the database. This was based on inputs from various stakeholders and was finalized with consensus from WHO and the participating SIHI hubs. Definitions of specific terms also provided additional guidance.

Table 2 ***Inclusion criteria and guiding definitions for the selection of community engagement materials***

Inclusion Criteria
1. Documented in reputable sources or can provide information/documentation for the assessment of validity
2. Articles published in the last 10 years or undocumented experiences active within the last 10 years

For peer review only - http://bmjopen.bmj.com/site/about/guidelines.xhtml

3. All community engagement criteria are met:
   a. Captures or documents experience on community engagement addressing a health need or social determinants of health
   b. Uses a participatory approach and active two-way communication using language appropriate for different actors and stakeholders
   c. Encourages collaboration/synergies and sharing of expertise with various stakeholders and sectors, mainly, but not limited to, marginalized groups to improve capacities
   d. Involves the community in the different phases of implementation of the intervention/strategy such as planning, context analysis, decision making, research, monitoring, evaluation and/or learning to ensure inclusive representation, maximum participation, and uncompromised consultation
   e. Builds and sustains trust within the community

      To simplify the assessment of trust, the following criteria based on the work of Di Napoli et al.[20], have been adopted. At least two of the four criteria must be met to indicate trust with the community:

      i. Presence of interest and competence in offering services that support the community's needs and allows the realization of the community members' aspirations
      ii. Community members are willing to participate in the improvement of the community through their effort of contribution of valuable resources
      iii. Community members find pleasure and meaning in spending their time participating
      iv. Community members expect that the engagement will improve future resources related to security, decision-making, participation, and achieving their goals

**Definitions of Terms**	
Communities	Groups of people who may or may not be spatially connected, but share common interests, concerns, or identities. These communities could be local, national or international, with specific or broad interests[21]
Community engagement	"The process of working collaboratively with and through groups of people affiliated by geographic proximity, special interest, or similar situations to address issues affecting the well-being of those people"[22]<br>"The process of developing relationships that enable stakeholders to work together to address health-related issues and promote wellbeing to achieve positive health impact and outcomes"[23]

For peer review only - http://bmjopen.bmj.com/site/about/guidelines.xhtml

Social determinants of health	"Non-medical factors that influence health outcomes". They are circumstances where "people are born, grow, work, live, and age, and the wider set of forces and systems shaping the conditions of daily life"[24]
Trust	"Positive expectations of community members toward the current and future opportunities they perceive in their local community, namely the place where they live and interact"[20]<br>Building purposeful and compassionate relationships through a resilient and community-competent health workforce that adapts to the needs and preferences of the people they serve[25]

Writing Case Summaries

A summary was written for each identified case including the project's name, implementing institution, number of years the project was implemented, implementation site, and health issues/topic addressed. The rationale, objectives, intervention, outcomes, lessons, challenges, and factors promoting and/or impeding CE were abstracted. Social innovations, if any, were included.

Compilation of Materials

All selected and created documents were uploaded to the project's Google Drive and kept in storage, pending migration to a WHO repository for the database, CELP, and CEWP.

Analysis and Identification of Common Themes

Content analysis of the summaries and other data extracted from the screened materials was done using open coding. Key ideas and nuances were identified and grouped into categories and themes. These were then used to tag and organize the materials in the database.

For peer review only - http://bmjopen.bmj.com/site/about/guidelines.xhtml

Development of the Community Engagement Learning Package

The CELP is a curation of CE lessons and tools presented as online (asynchronous) modules designed to capacitate learners on basic concepts, principles, and applications of CE, and explore best practice experiences in solving health problems and promoting health through CE. In-depth analysis done with the contents of the database identified important CE principles, practices, lessons, challenges, and barriers encountered in different contexts and regions. Existing CE frameworks, toolkits, and guides were also surveyed. Emerging themes and concepts were utilized as the basis for the development of the CELP. SMEs contributed to the contents of the CELP designed to be delivered in an online learning management system.

Initial outline and plans for the CELP were also vetted among the CEP WG, and stakeholders and partners who have extensive experience in engaging and mobilizing communities, both at the regional and global levels. Comments, critiques, suggestions, and recommendations that emerged from the series of vetting processes further shaped and enhanced the content of the learning package.

Development of the Community Engagement Workshop Package

The CEWP was developed as a complementary strategy to the CELP, highlighting important topics and practical activities that might be useful for participants to enhance their CE practice. It was initially designed for face-to-face engagements, but because of the restrictions brought about by the pandemic, the pilot implementation was done online. The package materials were made into a downloadable format that can be adapted in either online or face-to-face settings. Different iterations of the activity design were developed based on the different possible country contexts, utilizing the input from

For peer review only - http://bmjopen.bmj.com/site/about/guidelines.xhtml

SIHI networks and frontline responders engaging specific issues and populations - migrants, indigenous populations, people living with disabilities, women, elderly and youths.

Testing the Learning and Workshop Packages

Prototypes of the packages were tested among stakeholders, particularly community mobilizers, public health practitioners and other potential end-users.

An online platform was created to test the online learning package. Pilot participants were selected using criteria that facilitated the inclusion of different groups and were invited to undergo the online asynchronous training. Feedback from the participants were obtained through online evaluation forms and were used to guide the revision of the training design.

Pilot testing for the workshop package was conducted in two phases through an online video conferencing platform. The first phase was implemented among participants from the Philippines. The pilot run tested the regional applicability and impact of the materials and content. The second phase was conducted among a global set of participants, which tested its universal applicability and impact. In both phases, user experiences were collected and used to refine the packages.

Ethical Considerations

The development of the CEP did not entail participation of human subjects that requires ethical approval by the WHO Ethics Review Committee.[26] The collection of feedback from pilot participants is a regular mechanism to evaluate training. Informed consent was obtained before documenting CE practitioners' experiences and recording workshop

For peer review only - http://bmjopen.bmj.com/site/about/guidelines.xhtml

proceedings. Information about the project and its objectives and the extent of their participation were discussed.

Monitoring & Evaluation in Project Development

Regular internal SIHI and WHO reviews and consultative processes were facilitated to ensure that project deliverables met the needs of the end-users and fulfilled the objectives of the project.

Limitations in Conducting the CEP Activities/Process

All engagements and coordination for this project were done remotely using online platforms due to the restrictions brought about by the COVID-19 pandemic. The team ensured that participatory approaches were reinforced and the voices of CE practitioners were incorporated in the CEP.

RESULTS

Community Engagement Database

A database of experiences on CE was developed across public health in different settings. WHO and partners identified relevant resources that captured CE experiences, using the prescribed inclusion criteria. Materials in various formats (documents, videos, etc.) that highlighted the practices, lessons and challenges in working with the communities were compiled. The documents and related materials are in English, Spanish, and French. Summaries of documented CE cases are available in English.

For peer review only - http://bmjopen.bmj.com/site/about/guidelines.xhtml

Categories of Cases in the CE Database

There are 356 cases in the database (290 identified from published literature, 57 from grey literature, and nine from CE practitioner interviews) from all six WHO regions, categorized according to the health topic (Table 3). In addition, a total of 56 cases dealing with health emergencies were identified with 30 cases on COVID-19, 12 on Ebola, nine on environmental risk and disaster, and five on humanitarian crises.

Table 3 ***Distribution of Cases According to Health Topic and the WHO Regions***

Health Topic Category	No. of Cases per WHO Region						
	AFR	EMR	EUR	PAHO	SEAR	WPR	Total
Communicable Diseases	66	10	2	20	14	21	133
Primary Health Care	9	2	11	13	6	8	49
Maternal & Child Health	9	1	2	5	5	3	25
WASH	6	0	1	3	1	0	11
Sexual & Reproductive Health	3	2	2	4	1	2	14
Social Determinant of Health	1	5	13	27	7	3	56
Mental Health	0	3	1	5	1	4	14
NCDs	1	3	4	3	8	11	30
Nutrition	0	0	0	2	2	2	6
Others	3	0	5	3	5	2	18
**Total**	**98**	**26**	**41**	**85**	**50**	**56**	**356**

For peer review only - http://bmjopen.bmj.com/site/about/guidelines.xhtml

CE Practitioner Interviews

Seven CE practitioner interviews were conducted – five interviewees from AFRO, one each from PAHO and WPRO. These interviews identified nine unpublished CE experiences and explored CE strategies and dynamics and how that influenced the sustainability of health interventions.

Thematic Analysis

The case summaries were coded and analyzed, capturing themes from the rationale for CE, key insights, facilitating factors, and barriers. The documentation of the thematic analysis is available in a supplementary document in the database. Table 4 presents the thematic areas that emerged from the review of the cases.

Table 4 ***Summary of Themes from the Community Engagement Cases***

Rationale for Community Engagement	Contextual and health system challenges<br>Health and social goals<br>Mechanisms
Key Points and Insights	Community mobilization<br>Individual and community agency<br>Multi-stakeholder engagement<br>Multidirectional communication<br>Building on local capacity<br>Access, acceptability and adaptation<br>Inclusion<br>Sustainability<br>Participatory research<br>Basic principles
Facilitators of Community Engagement	Adapting the intervention<br>Applying participatory principles and approaches<br>Maximizing reach and access<br>Utilizing support mechanisms

For peer review only - http://bmjopen.bmj.com/site/about/guidelines.xhtml

Barriers to Community Engagement	Societal and contextual issues<br>Challenges with leadership<br>Weak health system<br>Challenges in encouraging and sustaining participation<br>Inadequate reach and access<br>Knowledge/information gaps<br>Lack of trust<br>Issues in communication<br>Inadequate or improper allocation of resources<br>Organizational and logistic problems<br>Challenges on the sustainability and generalizability of the project<br>Timing and duration of community engagement

Community Engagement Learning Package (CELP)

From the CE materials collected, the CELP was developed anchored on basic principles and standards of CE and grounded on actual experiences in working with communities in different contexts and settings. The CELP includes four self-instructional modules that participants may complete independently or as a ladder-type course. Each module presents basic frameworks and concepts of CE in relation to the theme of that module and are then tied to real world examples of CE in different contexts (see Table 5). Target learners include early to mid-level professionals and practitioners applying community engagement in their work who may come from various disciplines such as medical and health sciences, public health, public policy and administration, program management, social development and other social sciences. Students both at the undergraduate and postgraduate levels of any higher education institution, from various disciplines as mentioned above may also benefit from the modules.

For peer review only

For peer review only - http://bmjopen.bmj.com/site/about/guidelines.xhtml

Table 5 ***Modules of the Community Engagement Learning Package***

Module Title	Main framework/s used	Sample cases used
Module 1: Engaging and Mobilizing Communities for Health and Development	WHO community engagement framework for quality, people-centred and resilient health services[23]<br><br>Community engagement: a health promotion guide for universal health coverage in the hands of the people[27]	Setting health priorities in a community: a case example<br>Sousa et al., 2017[32]<br><br>Participatory learning and action to address type 2 diabetes in rural Bangladesh: a qualitative process evaluation<br>Morrison et al., 2019[33]<br><br>Community engagement in outbreak response: lessons from the 2014–2016 Ebola outbreak in Sierra Leone<br>Bedson et al., 2020[34]<br><br>'What works here doesn't work there': The significance of local context for a sustainable and replicable asset-based community intervention aimed at promoting social interaction in later life.<br>Wildman et al., 2019[35]
Module 2: Strengthening Health Systems through Community Engagement	Systems thinking for health systems strengthening[28]	Achieving UHC in Samoa through Revitalizing PHC and Reinvigorating the Role of Village Women Groups<br>Baghirov et al., 2019[36]
Module 3: Community Engagement in All-Hazards Emergency and Disaster Risk Management	Sendai framework for disaster risk reduction 2015–2030[29]<br><br>Health Emergency and Disaster Risk Management Framework[30]	Shifting Paradigms: Strengthening Institutions for Community-Based Disaster Risk Reduction and Management<br>Bawagan et al., 2015[37]

For peer review only - http://bmjopen.bmj.com/site/about/guidelines.xhtml

Module 4: Community Engagement as a Driver for Achieving Health Equity and Community Resilience	Minimum Quality Standards and Indicators for Community Engagement[31]	Integrated vector control of Chagas disease in Guatemala: a case of social innovation in health<br>Castro-Arroyave et al., 2020[38]

The pilot participants found the CELP to be comprehensive in terms of content and with a user-friendly format. They appreciated how other concepts in public health were linked to CE. They suggested more practical applications and specific how-to's, and assessment activities with immediate feedback. These were all taken into consideration in the revision of the modules.

Community Engagement Workshop Package (CEWP)

The CEWP provides tools and templates for identifying other CE experiences in a workshop format. The contents are similar to the CELP, with a special focus on documenting "new" CE experiences and a walk-through of using and submitting case studies for the CE database. The target participants are practitioners who are interested in sharing their CE experiences. The CEWP allows the continuous collection of evidence and discussions with stakeholders on CE principles, practices, and frameworks. These resources will be cataloged, categorized, and used to update the database and the learning and workshop packages.

Participants and observers of the CEWP pilot were satisfied with the introduction and ice-breaking activities which set the stage for conducive training sessions. Participants also expressed satisfaction on the content, pointing out that the workshop addressed aspects of CE not previously considered. The topics of the training were noted to be far-reaching,

For peer review only - http://bmjopen.bmj.com/site/about/guidelines.xhtml

covering several CE frameworks, with good video presentations. Participants were able to relate the lessons and case studies to their experiences. They pointed out a few areas of improvement, including the need for adequate time to study the cases prior to the synchronous online sessions and more breakout sessions for participants to raise issues and ensure more diverse voices and opinions. They also recommended that the frameworks need to further emphasize listening and understanding community perspectives right from the start of the engagement.

DISCUSSION

The CEP and its development showcase innovative elements in the project design, the human resources involved and way of working, and the interrelationships of the different CEP components.

The CEP conceptualization and design involved broad consultations and co-creation with a community of diverse teams of WHO, SIHI hubs, UNICEF, and other implementing partners, and frontline responders. The process and products of the package were vetted among stakeholders and partners at the regional and global levels. In addition, community practitioners were consulted regarding the screening criteria of cases to be included in the database, shared undocumented CE practices, and participated in the pilots of the learning and workshop packages to provide user feedback. This multi-stakeholder consultative processes allowed for the creation of a grounded, contextualized, relevant and integrated package.

Working on the CEP project during the COVID-19 pandemic did not deter the WHO and SIHI from intensifying collaboration. The use of online platforms enabled the team to engage and mobilize relevant resources and develop the CEP components despite the

For peer review only - http://bmjopen.bmj.com/site/about/guidelines.xhtml

absence of face-to-face consultations and other limitations. Creative use of online platforms was also maximized for the different components of the CEP (e.g., online database, online modules) while still providing templates for possible face-to-face delivery, allowing for flexibility in engagement methods.

The three components of the CEP feed into each other. The thematic analysis of the materials in the CE database guided the design of the CELP and CEWP. Selected cases were also used to reinforce and provide real-world application to the CE frameworks and related concepts in the online modules. The CEWP facilitates the discussion of CE principles and practices among practitioners and the collection of new information for updating the database and CELP with "new" CE experiences.

The merit of the current CEP project over existing documentation is that the CEP is broad-based - not limited to health emergencies, but includes other public health and social developmental activities such as routine immunization, neglected tropical diseases, city and urban development, nutritional interventions and disaster risk management, among others.

An operational challenge during the documentation was the language barrier. The cases were limited to English, French and Spanish. Future researchers can explore relevant documented and undocumented experiences in other languages, which will make the database more comprehensive and unifying at the same time.

CONCLUSION

The design of the CEP emphasized interrelationships among its components – CE database, learning package and workshop package. The CELP contents were taken from the comprehensive thematic analysis of the database. The CEWP facilitates the

documentation of "new" CE experiences and their nuances, ensuring timely updates of the database by CE practitioners themselves.

Most of the cases included in the CEP database presented key insights on CE including its basic principles and the role of individual and community agency, building on local capacity, multi-directional communication, inclusion, and multi-stakeholder engagement. Barriers to CE including issues of access, acceptability and adoption in the setting of weak health systems and societal issues were also identified. The learning and workshop packages were then developed to guide health professionals and other stakeholders based on these grounds.

The development of the CEP was the work of multiple global stakeholders providing synergistic contributions and bridging silos. The description of the CEP methodology will allow replication, provide transparency into the development of the CEP and present lessons learned during the development of a robust and harmonized package.

ACKNOWLEDGEMENTS

The authors would like to thank Dr. Nedret Emiroglu, **Director, WHO Country Readiness Strengthening Department, WHO**; the **WHO CEP Working Group members and resource persons**: Qudsia Huda, Ankur Rakesh, Sohel Saikat, Saqif Mustafa, Samar Elfeky, Mervat Gawrgyous, Suvajee Good, Alex Camacho, Julienne Ngoundoung Anoko, Aphaluck Bhatiasevi, Faten Ben Abdelaziz, Ana Gerlin Hernandez Bonilla, Mihai Mihut, Nicole Valentine, Mary Manandhar, Dayo Spencer-Walters, Philippe Eric Gasquet, Joao Jose Salavessa Rangel De Almeida, Nina Gobat, Redda Seifeldin, Sameera Suri, Renee Christensen, Melinda Frost, Simon Van Woerden, Cristiana Salvi, Leonardo Palumbo, Aminata Grace Kobie, Anna Coates, Sonja Caffe, Gerry Eijkemans, Orielle Solar

For peer review only - http://bmjopen.bmj.com/site/about/guidelines.xhtml

Hormazabal, Tonia Rifaey, Peggy Edmond Hanna, Godfrey Yikii, Dalia Samhouri, Supriya Bezbaruah, Kira Fortune and UNICEF resource persons Rania Elessawi, Naureen Naqvi, Ana Puri; the **RCCE Collectives Services colleagues** from the International Federation of the Red Cross and Red Crescent Societies (IFRC), UNICEF, Global Outbreak Alert and Response Network (GOARN) and WHO; and the **CEP project staff, consultants and research associates:** Erlyn Sana, Nina Castillo-Carandang, Gladys Armada, Jennel Pimentel, Justin Bryan Maranan, Nathalia Palma, Philippe Galban, Clarence Diaz, Eric David Ornos, Celina Gonzales, Samuel Tristan Vinluan, Ma. Pamela Tagle, Linda Mipando-Nyondo, Deborah Nyirenda, Katusha de Villiers, José Alejandro Carias, Perla Simons Morales, Sandra Barahona, Karla Zúniga, and Milena Bautista.

CONTRIBUTORSHIP

YB, MDPL, JRBC, JDMA, UEO, AOJ, and NRJ conceptualized and designed this work. JRBC, JDMA, PMPT, ARU, MF, MIE, JA, DM, BKM, OIE, ON, LA, NG, CNA, BB, EC, EN, VNK, and GMK gathered and analyzed data. YB, UEO, SAO, JRBC, JDMA, and PMPT drafted the manuscript. All authors reviewed, edited, and approved the final version of the manuscript.

COMPETING INTERESTS

There are no competing interests for any author.

FUNDING

The development of the WHO Community Engagement Package was funded by the World Health Organization. The Social Innovation in Health Initiative (SIHI) is funded by TDR, the Special Programme for Research and Training in Tropical Diseases co-sponsored by UNICEF, UNDP, the World Bank, and WHO. TDR receives additional funding from the Swedish International Development Cooperation Agency (Sida), to support SIHI.

Grant/Award Number: N/A

For peer review only - http://bmjopen.bmj.com/site/about/guidelines.xhtml

DATA SHARING/DATA AVAILABILITY

Data are available upon reasonable request.

ETHICS APPROVAL STATEMENT

This study does not involve human participants.

REFERENCES

1. WHO. The impact of the COVID-19 pandemic on noncommunicable disease resources and services: results of a rapid assessment [Internet]. 2020 Sep [cited 2021 Oct 19] p. 22. Available from: https://www.who.int/publications-detail-redirect/9789240010291

2. Zawawi A, Alghanmi M, Alsaady I, Gattan H, Zakai H, Couper K. The impact of COVID-19 pandemic on malaria elimination. Parasite Epidemiol Control. 2020;11(November):e00187.

3. Ters LET. COVID-19 policies : Remember measles Restart science stronger after COVID-19 Uphold the nuclear weapons test moratorium. 2020;369(6501):261–3.

4. Sun X, Samba TT, Yao J, Yin W, Xiao L, Liu F, et al. impact of the Ebola outbreak on routine immunization in western area, Sierra Leone - A field survey from an Ebola epidemic area. BMC Public Health. 2017;17(1):1–6.

5. WHO. COVID-19 Global Risk Communication and Community Engagement Strategy – interim guidance [Internet]. 2020 [cited 2021 Oct 19]. Available from: https://www.who.int/publications-detail-redirect/covid-19-global-risk-communication-and-community-engagement-strategy

6. IFRC. From words to action: Towards a community-centred approach to preparedness and response in health emergencies [Internet]. 2019 [cited 2021 Oct 19]. Available from: https://www.gpmb.org/annual-reports/overview/item/from-words-to-action-towards-a-community-centred-approach-to-preparedness-and-response-in-health-emergencies

7. Gillespie AM, Obregon R, Asawi RE, Richey C, Manoncourt E, Joshi K, et al. Social Mobilization and Community Engagement Central to the Ebola Response in West Africa: Lessons for Future Public Health Emergencies. Glob Health Sci Pract. 2016 Dec 23;4(4):626–46.

8. Wright K, Parker M. In emergencies, health research must go beyond public engagement toward a true partnership with those affected. Nat Med. 2020 Mar;26(3):308–9.

For peer review only - http://bmjopen.bmj.com/site/about/guidelines.xhtml

9. O'Mara-Eves A, Brunton G, Oliver S, Kavanagh J, Jamal F, Thomas J. The effectiveness of community engagement in public health interventions for disadvantaged groups: a meta-analysis. BMC Public Health. 2015 Feb 12;15(1):129.

10. UN OCHA. Workshop Report Final_Community Engagement in Humanitarian Action.pdf [Internet]. ReliefWeb. 2016 [cited 2021 Oct 19]. Available from: https://reliefweb.int/sites/reliefweb.int/files/resources/Workshop%20Report%20Final_Community%20Engagement%20in%20Humanitarian%20Action.pdf

11. IFRC. How your community can prevent the spread of COVID-19. 2020;19(March):1–5.

12. Bedford J, Chitnis K, Webber N, Dixon P, Limwame K, Elessawi R, et al. Community Engagement in Liberia: Routine Immunization Post-Ebola. J Health Commun. 2017;22(0):81–90.

13. Habib MA, Soofi S, Cousens S, Anwar S, Haque N ul, Ahmed I, et al. Community engagement and integrated health and polio immunisation campaigns in conflict-affected areas of Pakistan: a cluster randomised controlled trial. Lancet Glob Health. 2017;5(6):e593–603.

14. Mayhew SH, Kyamusugulwa PM, Bindu KK, Richards P, Kiyungu C, Balabanova D. Responding to the 2018 – 2020 Ebola Virus Outbreak in the Democratic Republic of the Congo : Rethinking Humanitarian Approaches. 2021;1731–47.

15. Oleribe OO, Salako BL, Ka MM, Akpalu A, McConnochie M, Foster M, et al. Ebola virus disease epidemic in West Africa: Lessons learned and issues arising from West African countries. Clin Med J R Coll Physicians Lond. 2015;15(1):54–7.

16. Mason C, Barraket J, Friel S, O'Rourke K, Stenta CP. Social innovation for the promotion of health equity. Health Promot Int. 2015;30:ii116–25.

17. Halpaap BM, Tucker JD, Mathanga D, Juban N, Awor P, Saravia NG, et al. Social innovation in global health: sparking location action. Lancet Glob Health. 2020;8(5):e633–4.

18. DiClemente R, Nowara A, Shelton R, Wingood G. Need for innovation in public health research. Am J Public Health. 2019;109:S117–20.

19. Omoleke SA, Bayugo YV, Oyene UE, Abrahams J, Gobat N, Good S, et al. WHO Community Engagement Package: A Reinforcement of an Inclusive Approach to Global Public Health. Int J Epidemiol Health Sci [Internet]. 2021 Jul 1 [cited 2021 Oct 12];2(7). Available from: http://www.ijehs.com/article_244835.html

20. Di Napoli I, Dolce P, Arcidiacono C. Community Trust: A Social Indicator Related to Community Engagement. Soc Indic Res. 2019 Sep 1;145(2):551–79.

For peer review only - http://bmjopen.bmj.com/site/about/guidelines.xhtml

21. WHO. The 7th Global Conference on Health Promotion [Internet]. 2009 [cited 2021 Oct 12]. Available from: https://www.who.int/teams/health-promotion/enhanced-wellbeing/seventh-global-conference/community-empowerment

22. DHHS. Principles of Community Engagement [Internet]. 2nd ed. NIH; 2011. Available from: http://www.atsdr.cdc.gov/communityengagement/pdf/PCE_Report_508_FINAL.pdf

23. WHO. WHO community engagement framework for quality, people-centred and resilient health services. 2017. Available from: https://apps.who.int/iris/handle/10665/259280. License: CC BY-NC-SA 3.0 IGO

24. WHO. Health in the post-2015 development agenda : need for a social determinants of health approach Joint statement of the UN Platform on Social Determinants of Health. Jt Statement UN Platf Soc Determinants Health. 2015;1–18.

25. WHO. Enhanced Capacity Building Training for Frontline Staff on Building Trust and Communication. 2015;(July).

26. WHO. Ethics Review Committee Frequently Asked Questions [Internet]. 2019 [cited 2022 Jan 10]. Available from: https://extranet.who.int/ercweb/faq.php#fq6

27. WHO. Community engagement: a health promotion guide for universal health coverage in the hands of the people. 2020. Available from: https://apps.who.int/iris/handle/10665/334379. License: CC BY-NC-SA 3.0 IGO

28. Don DS and Taghreed A. Systems thinking for health systems strengthening. Alliance for Health Policy and Systems Research, WHO. 2009. Available from: https://apps.who.int/iris/bitstream/handle/10665/44204/9789241563895_eng.pdf?sequence=1

29. United Nations. Sendai framework for disaster risk reduction 2015–2030. 2015. Available from: https://www.preventionweb.net/files/43291_sendaiframeworkfordrren.pdf

30. WHO. Health Emergency and Disaster Risk Management Framework. 2019. Available from: https://www.who.int/hac/techguidance/preparedness/health-emergency-and-disaster-risk-management-framework-eng.pdf Licence: CC BY-NC-SA 3.0 IGO.

31. UNICEF. Minimum quality standards and indicators in community engagement [Internet]. 2020 [cited 2021 Oct 19]. Available from: https://www.unicef.org/mena/reports/community-engagement-standards

32. Sousa FAMR, Goulart MJG, Braga AMS, Medeiros CMO, Rego DCM, Vieira FG, et al. Setting health priorities in a community: a case example. Rev Saude Publica. 2017; 51:11. Available from: https://www.ncbi.nlm.nih.gov/pmc/articles/PMC5336317/pdf/0034-8910-rsp-S1518-87872017051006460.pdf

For peer review only - http://bmjopen.bmj.com/site/about/guidelines.xhtml

33. Morrison J, Akter K, Jennings HM, et al. Participatory learning and action to address type 2 diabetes in rural Bangladesh: a qualitative process evaluation. BMC Endocr Disord. 2019; 19,118 https://doi.org/10.1186/s12902-019-0447-3

34. Bedson J, Jalloh MF, Pedi D, et al. Community engagement in outbreak response: lessons from the 2014–2016 Ebola outbreak in Sierra Leone. BMJ Global Health. 2020; 5, Article e002145. doi:10.1136/ bmjgh-2019-002145

35. Wildman JM, Valtorta N, Moffatt S & Hanratty B. 'What works here doesn't work there': The significance of local context for a sustainable and replicable asset-based community intervention aimed at promoting social interaction in later life. Health Soc Care Community. 2019; 27, 1102– 1110. https://doi.org/10.1111/hsc.12735

36. Baghirov R, Ah-Ching J & Bollars C. Achieving UHC in Samoa through Revitalizing PHC and Reinvigorating the Role of Village Women Groups. Health Systems & Reform. 2019; 5:1, 78-82, DOI: 10.1080/23288604.2018.1539062

37. Bawagan AB, Polotan-dela Cruz L, Felizco MS, Tan MJ, Wamil M & Germar JS. Shifting Paradigms: Strengthening Institutions for Community-Based Disaster Risk Reduction and Management. Quezon City: UP College of Social Work and Community Development. 2015. Available from: http://iskwiki.upd.edu.ph/flipbook/viewer/?fb=2012-79319-U.P.-SHI&fbclid=IwAR1CKfUX_3RcdAoJiq9-4N0f6o7zCvvANoy7EnrAoMKn-rKik9EyDM4aXas#page-1

38. Castro-Arroyave D, Monroy MC & Irurita MI. Integrated vector control of Chagas disease in Guatemala: a case of social innovation in health. Infect Dis Poverty. 2020; 9:25. https://doi.org/10.1186/s40249-020-00639-w

For peer review only - http://bmjopen.bmj.com/site/about/guidelines.xhtml

Figure Legend

Figure 1. WHO Community Engagement Package Components and Relationships

For peer review only

For peer review only - http://bmjopen.bmj.com/site/about/guidelines.xhtml

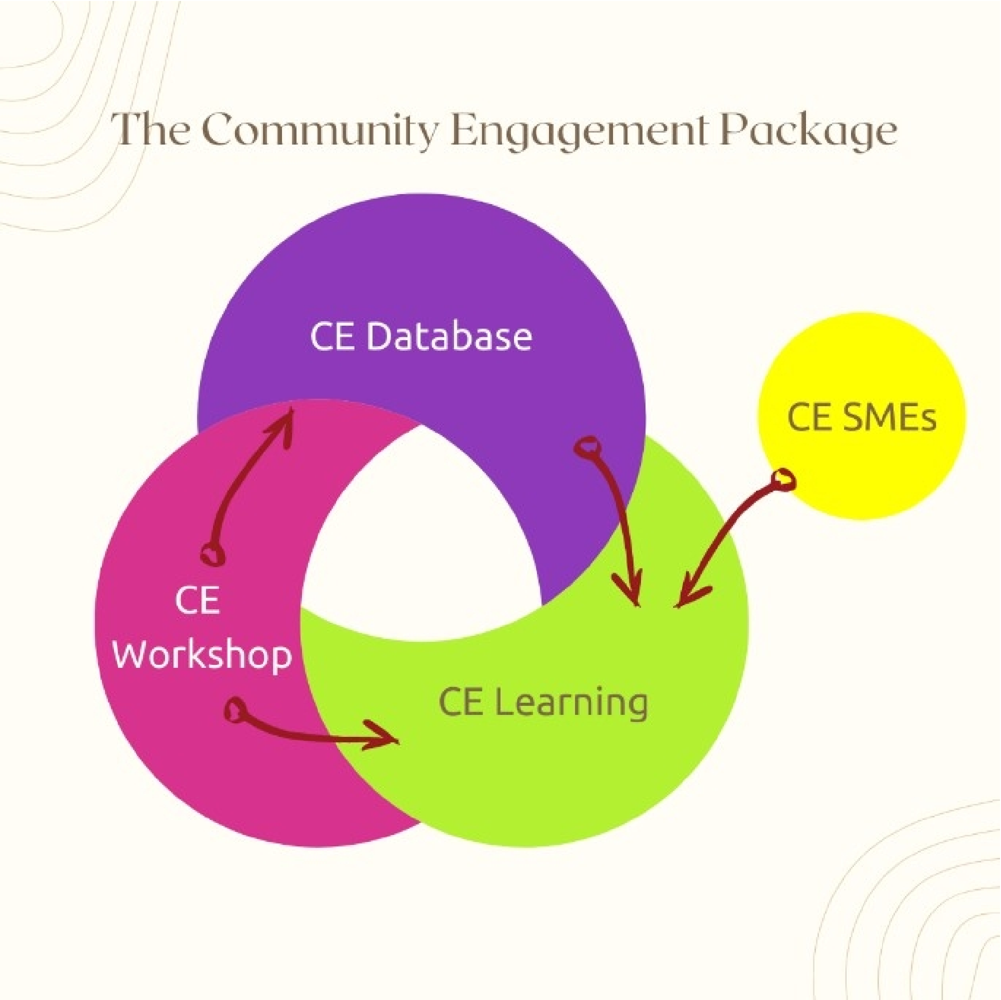

181x180mm (96 x 96 DPI)

BMJ Open

A Description of Global Innovative Methods in Developing the WHO Community Engagement Package

Journal:	*BMJ Open*
Manuscript ID	bmjopen-2022-063144.R1
Article Type:	Original research
Date Submitted by the Author:	04-May-2022

Complete List of Authors:	Bayugo, Yolanda; World Health Organization, Country Readiness Strengthening<br>Labarda, Meredith; University of the Philippines Manila Department of Clinical Epidemiology; University of the Philippines Manila School of Health Sciences<br>Cruz, Jose Rene Bagani; University of the Philippines Manila<br>Mier-Alpano, Jana Deborah; University of the Philippines Manila<br>Tiangco, Pauline Marie; University of the Philippines Manila<br>Oyene, Ukam; World Health Organization<br>Omoleke, Semeeh; World Health Organization, Field Presence; World Health Organization, Country Readiness Strengthening<br>Ulitin, Allan; University of the Philippines Manila,<br>Ong Jr., Alberto; Alliance for Improving Health Outcomes<br>Fajardo, Marvinson; University of the Philippines Manila<br>Echavarria, Maria Isabel; Centro Internacional de Entrenamiento e investigaciones médicas (CIDEIM); Universidad Icesi<br>Alger, Jackeline; Hospital Escuela, ; Instituto de Enfermedades Infecciosas y Parasitologia Antonio Vidal, NA<br>Mathanga, Don; University of Malawi College of Medicine<br>Msiska, Barwani ; Kamuzu University of Health Sciences,<br>Ekwunife, Obinna Ikechukwu; Nnamdi Azikiwe University, Clinical Pharmacy and Pharmacy Management; Nnamdi Azikiwe University, Department of Clinical Pharmacy and Pharmacy Management<br>Nwaorgu, Obioma; Nnamdi Azikiwe University; Nnamdi Azikiwe University, Department of Parasitology and Entomology<br>Abella Lizcano, Lorena; Centro Internacional de Entrenamiento e investigaciones médicas (CIDEIM); Universidad Icesi<br>gomez quenguan, Natalia; Centro Internacional de Entrenamiento e investigaciones médicas (CIDEIM); Universidad Icesi<br>Nieto Anderson, Claudia; SIHI Honduras Hub,<br>Beltran, Briana; Centro de educación medica continua Honduras,<br>Carcamo Rodriguez, Elsy; Independent Researcher, Francisco Morazán; Independent Researcher, Francisco Morazán<br>Núñez, Eduardo; Universidad Católica de Honduras Nuestra Señora Reina de la Paz Facultad de Ciencias de la Salud, Facultad de Ciencias Médicas; Hospital General Santa Teresa, Cirugía General<br>Nkosi-Kholimeliwa, Vera; Evangelical Lutheran Development Services<br>Mwafulirwa-Kabaghe, Glory; Joyful Motherhood, Maternal and Newborn care<br>Juban, Noel; University of the Philippines Manila Department of Clinical Epidemiology

**Primary Subject Heading**:	Global health
Secondary Subject Heading:	Public health
Keywords:	PUBLIC HEALTH, SOCIAL MEDICINE, PRIMARY CARE

SCHOLARONE™
Manuscripts

I, the Submitting Author has the right to grant and does grant on behalf of all authors of the Work (as defined in the below author licence), an exclusive licence and/or a non-exclusive licence for contributions from authors who are: i) UK Crown employees; ii) where BMJ has agreed a CC-BY licence shall apply, and/or iii) in accordance with the terms applicable for US Federal Government officers or employees acting as part of their official duties; on a worldwide, perpetual, irrevocable, royalty-free basis to BMJ Publishing Group Ltd ("BMJ") its licensees and where the relevant Journal is co-owned by BMJ to the co-owners of the Journal, to publish the Work in this journal and any other BMJ products and to exploit all rights, as set out in our licence.

The Submitting Author accepts and understands that any supply made under these terms is made by BMJ to the Submitting Author unless you are acting as an employee on behalf of your employer or a postgraduate student of an affiliated institution which is paying any applicable article publishing charge ("APC") for Open Access articles. Where the Submitting Author wishes to make the Work available on an Open Access basis (and intends to pay the relevant APC), the terms of reuse of such Open Access shall be governed by a Creative Commons licence – details of these licences and which Creative Commons licence will apply to this Work are set out in our licence referred to above.

Other than as permitted in any relevant BMJ Author's Self Archiving Policies, I confirm this Work has not been accepted for publication elsewhere, is not being considered for publication elsewhere and does not duplicate material already published. I confirm all authors consent to publication of this Work and authorise the granting of this licence.

For peer review only

A Description of Global Innovative Methods in Developing the WHO Community Engagement Package

Yolanda Bayugo[1], Meredith del Pilar-Labarda[2,3], Jose Rene Bagani Cruz[3], Jana Deborah Mier-Alpaño[3], Pauline Marie P. Tiangco[3], Ukam Ebe Oyene[1], Semeeh Akinwale Omoleke[1], Allan R. Ulitin[3], Alberto Ong Jr[3], Marvinson Fajardo[3], María Isabel Echavarría[4], Jackeline Alger[5], Don Mathanga[6], Barwani Khaura Msiska[6], Obinna I. Ekwunife[7], Obioma Nwaorgu[7], Lorena Abella[4], Natalia Gómez[4], Claudia Nieto-Anderson[8], Briana Beltran[9], Elsy Cárcamo[10], Eduardo Núñez[10], Vera Nkosi-Kholimeliwa[11], Glory Mwafulirwa-Kabaghe[12], and Noel R. Juban[3,13]

[1] Country Readiness Strengthening Department, Health Emergencies Programme, World Health Organization, Geneva, Switzerland

[2] Department of Medicine, School of Health Sciences, University of Philippines Manila, Philippines

[3] Social Innovation in Health Initiative Philippines, University of the Philippines Manila, Philippines

[4] Centro Internacional de Entrenamiento e Investigaciones Médicas (CIDEIM), Colombia

[5] Facultad de Ciencias Médicas, Universidad Nacional Autónoma de Honduras (UNAH), Tegucigalpa, Honduras

[6] Social Innovation in Health Initiative Malawi, Kamuzu University of Health Sciences, Blantyre, Malawi

[7] Social Innovation in Health Initiative Nigeria, Nnamdi Azikiwe University, Awka, Nigeria

[8] Dirección de Investigación Científica, Universidad Nacional Autónoma de Honduras (UNAH), Tegucigalpa, Honduras

[9] Centro Nacional de Educación Médica Continua, Colegio Médico de Honduras, Tegucigalpa, Honduras

[10] Universidad Nacional Autónoma de Honduras (UNAH), Tegucigalpa, Honduras

[11] Evangelical Lutheran Development Services (ELDS), Malawi

[12] Joyful Motherhood, Lilongwe, Malawi

[13] Department of Clinical Epidemiology, College of Medicine, University of the Philippines Manila, Philippines

Corresponding author: Meredith del Pilar-Labarda, mdlabarda@up.edu.ph

Word count: 3,285

For peer review only

ABSTRACT

Objectives Development of a Community Engagement Package (CEP) composed of (1) database of community engagement (CE) experiences from different contexts, (2) CE learning package of lessons and tools presented as online modules, and (3) CE workshop package for identifying CE experiences to enrich the CE database and ensure regular update of learning resources. The package aims to guide practitioners to promote local action and enhance skills for CE.

Setting and Participants The packages were co-created with diverse teams from WHO, SIHI, UNICEF, community practitioners, and other partners providing synergistic contributions and bridging existing silos.

Methods The design process of the package was anchored on CE principles. Literature search was performed using standardized search terms through global and regional databases. Interviews with CE practitioners were also conducted.

Results A total of 356 cases were found to fit the inclusion criteria and proceeded to data extraction and thematic analysis. Themes were organized according to rationale, key points and insights, facilitators of CE, and barriers to CE. Principles and standards of CE in various contexts served as a foundation for the CE learning package. The package comprises four modules organized by major themes such as mobilizing communities, strengthening health systems, CE in health emergencies, and CE as a driver for health equity.

Conclusion After pilot implementation, tools and resources were made available for training and continuous collection of novel CE lessons and experiences from diverse socio-geographical contexts.

For peer review only - http://bmjopen.bmj.com/site/about/guidelines.xhtml

STRENGTHS AND LIMITATIONS OF THIS STUDY

- The WHO Community Engagement Package (CEP) was co-created with a community of diverse teams of WHO, Social Innovation in Health Initiative hubs, UNICEF, partners organizations, and community practitioners that provided synergistic contributions in promoting best community engagement (CE) practices across the board.
- This project fills a need for a harmonized CE documentation package for training based on different local contexts and with a broad range of health and social development activities including health emergencies, routine immunization, neglected tropical diseases, city and urban development, nutritional interventions, and disaster risk management.
- The CE cases identified were limited to those in English, French, and Spanish. Future researches can explore relevant documented and undocumented experiences in other languages.
- The CEP was developed and tested primarily through online environments and might need adjustment for in-person implementation.

INTRODUCTION

There is an increasing necessity to redouble efforts using innovative approaches to bolster community engagement (CE) in the global health setting. Emergencies, including the COVID-19 pandemic, severely disrupted prevention and treatment services for non-communicable diseases (NCDs), malaria and other interventions.[1-4] This has compounded health inequities and widened the gap across populations. The complexities brought about by these health problems make community participation in co-creating innovative solutions to these challenges even more critical. The shift to people-centred approaches as highlighted in the revised WHO risk communication and community engagement (RCCE) strategy,[5, 6] is imperative as CE can make a considerable difference in health outcomes and capacitate communities to deal with

For peer review only - http://bmjopen.bmj.com/site/about/guidelines.xhtml

health challenges and their determinants.[7-9] The response to the Nepal earthquake and similar experiences made clear that people-centred design and leadership in addressing problems facilitate more efficient use of resources, strengthen coordination and build local capacities.[10] The World Health Organization (WHO), United Nations Children's Fund (UNICEF) and development partners support CE with resource mobilization, information, and trainings with various outcomes and competencies.[11] However, there is no harmonized CE documentation package based on local contexts for training. This project was initiated to guide health practitioners in promoting local action, and to facilitate involvement, training, and synergies across health and development sectors to achieve collective outputs and outcomes.[12-15] It responds to the need to invest in effective social innovations grounded on CE, which utilize bottom-up approaches and draw on strengths of individuals, communities, and institutions, while promoting synergies across sectors.[16-18]

The WHO Community Engagement Package

The WHO Department of Country Readiness Strengthening conceptualized and initiated the Community Engagement Package (CEP) project based on consultations within WHO Regional Offices and Headquarters. The CEP project[19] developed a database of CE experiences, a CE learning package (CELP), and a CE workshop package (CEWP) based on a broad scope of CE experiences in different settings. The compiled cases can guide program managers, CE practitioners, in-service medical and non-medical trainees, non-governmental organizations (NGO) staff, and multidisciplinary teams to sharpen their skills in the CE approach.

For peer review only - http://bmjopen.bmj.com/site/about/guidelines.xhtml

CEP Project Design and Components

The design of the CEP involved the creation of a database of relevant CE cases. These cases were categorized and analyzed, and themes and concepts were used to develop the CELP with contributions from CE subject matter experts (SMEs). The CEWP was designed to document "newer" CE experiences that can be incorporated into the database, ensuring regular updates of the learning resources (see Figure 1). Table 1 summarizes the three components of the CEP.

Figure 1. WHO Community Engagement Package Components and Relationships

Table 1 ***Descriptions of the Components of the WHO Community Engagement Package***

Community Engagement Database	Organized collection of data and documentation of community engagement experiences, practices, and approaches in different regions and contexts.
Community Engagement Learning Package	Curation of community engagement lessons and tools presented as online (asynchronous) modules designed to capacitate learners on basic concepts, principles, and applications of community engagement, and explore best practice experiences in solving health problems and promoting health through community engagement.
Community Engagement Workshop Package	Provides tools and templates for identifying community engagement experiences in a workshop format. The contents are similar to the Community Engagement Learning Package, with a special focus on documenting "new" CE experiences and their nuances, and a walk-through of using and submitting case studies for the CE database.

Given the uniqueness, relevance, and value of the harmonized CEP in the context of health emergencies and the overall global health sphere, this paper seeks to document

For peer review only - http://bmjopen.bmj.com/site/about/guidelines.xhtml

the processes and the innovative ways by which the CEP was developed at the height of COVID-19 restrictions.

METHODS

Patient and Public Involvement

The conceptualization, design, and conduct of the CEP involved participation and co-creation among colleagues and potential end users in the WHO, SIHI hubs, UNICEF and other implementing partners, and community practitioners and frontline responders.

CEP Human Resource Infrastructure and Way of Working

The overall project methodology was anchored on CE principles and processes. Colleagues in WHO (Headquarters and regions) participated in the CEP project. The Social Innovation in Health Initiative (SIHI) global network contributed substantially to the realization of the CEP.

WHO CEP Working Group

The design of the CEP project came about after consultations with WHO colleagues involved in CE work, bringing in experiences of WHO working with communities in different contexts and settings.[19] These colleagues work in different thematic areas: health promotion, social determinants of health, health systems, disaster risk reduction, risk communication, healthy cities, community readiness and resilience, and population-based focused work. As the CEP design was drafted, a working group (WG) was established to provide technical advice and CE resources related to their respective

For peer review only - http://bmjopen.bmj.com/site/about/guidelines.xhtml

areas of work. Regular WG meetings were conducted to ensure that they had updated information and an opportunity to provide feedback to improve the package. Some members of the WG also participated as resource persons in the CELP.

The WG also consulted and regularly updated the RCCE Collective Services, which is composed of WHO, UNICEF, International Federation of Red Cross and Red Crescent Societies (IFRC) and Global Outbreak Alert and Response Network (GOARN). UNICEF provided inputs regarding training.

SIHI Global Network

The SIHI Philippines Hub is the main implementing agency of the project. It is part of the SIHI global network of research hubs and other partners supported by TDR, the Special Programme for Research and Training in Tropical Diseases. SIHI hubs have expertise and experience documenting social innovations from communities and communicating these innovations with stakeholders.

Led by the SIHI Philippines, the SIHI hubs based in Colombia, Honduras, Malawi, Nigeria, and South Africa also participated in this project. Together, they gathered published and grey literature on CE and were involved in the development of the search terms and selection criteria, case abstracts and identification of themes. SIHI Philippines spearheaded the development of the prototype learning and workshop packages and facilitated regular virtual meetings with the other hubs and WHO staff for updates and consultation.

For peer review only - http://bmjopen.bmj.com/site/about/guidelines.xhtml

Development of the Components of the CEP

The development of the components of the CEP can be characterized as iterative, collaborative and comprehensive and can be considered "community engagement in practice."

Development of the Community Engagement Database

The CE database is an organized collection of data and documentation of CE practices, experiences, and approaches used in different regions and contexts. Systematic search was done to gather and organize these, integrating multi-stakeholder and consultative approaches across the SIHI global network and key partners from WHO.

Search for Materials on Community Engagement

This phase identified materials that document experiences about CE in programs that address health or the social determinants of health. The search procedures were developed and co-created with SIHI hubs and the WHO using the "system lens" principles and a bottom-up approach. Methods were refined as feedback was collected during implementation.

A standard procedure was prescribed for literature search to ensure the quality of cases found and maximize use of search platforms. For published literature (i.e. case reports/series, review articles, research papers, journal articles), searches in PubMed, Google Scholar, Hinari, Research Gate, Scopus, Embase, LILACS were conducted. Other significant local and regional repositories were also explored.

The following standard search terms were used:

For peer review only - http://bmjopen.bmj.com/site/about/guidelines.xhtml

- ("social" OR "community" OR "stakeholder") AND ("engagement" OR "partnerships" OR "participation" OR "action" OR "involvement" OR "empowerment")
- ("social" OR "community") AND ("ownership" OR "relations" OR "outreach")
- "community-based participatory research"
- "population health management"
- "community-directed intervention"

These terms were also translated to French and Spanish and additional terms for a geographic location were also added to focus searches in these areas.

For grey literature (i.e. newsletters, unpublished reports or limited distribution, theses, conference papers/presentations, books, and others) general search engines were used and academic and professional networks were tapped. Materials in languages other than English were included, with interpretation assistance from the SIHI network. Audiovisual materials were collected from credible organizational partners of WHO and SIHI, sources recommended by these organizations, and verified social media accounts and websites.

Interviews, surveys, and correspondence with CE practitioners were facilitated to identify undocumented CE practices. Academic and professional networks of the SIHI network, WHO, and partners were engaged in identifying undocumented CE practices for inclusion. Virtual communication technologies were utilized because of travel restrictions. Recordings or transcripts were obtained for documentation. The reviews were conducted by the project staff and SIHI hubs in coordination with the WG.

For peer review only - http://bmjopen.bmj.com/site/about/guidelines.xhtml

Following PRISMA's recommended process flow, materials collected were screened initially through the title and abstract, when available. These were then assessed based on the selection criteria.

Selection Criteria

A set of criteria (Table 2) was developed to standardize relevant CE cases that were entered into the database. This was based on inputs from various stakeholders and was finalized with consensus from WHO and the participating SIHI hubs. Definitions of specific terms also provided additional guidance.

Table 2 ***Inclusion criteria and guiding definitions for the selection of community engagement materials***

Inclusion Criteria
1. Documented in reputable sources or can provide information/documentation for the assessment of validity
2. Articles published in the last 10 years or undocumented experiences active within the last 10 years

For peer review only - http://bmjopen.bmj.com/site/about/guidelines.xhtml

3. All community engagement criteria are met:
   a. Captures or documents experience on community engagement addressing a health need or social determinants of health
   b. Uses a participatory approach and active two-way communication using language appropriate for different actors and stakeholders
   c. Encourages collaboration/synergies and sharing of expertise with various stakeholders and sectors, mainly, but not limited to, marginalized groups to improve capacities
   d. Involves the community in the different phases of implementation of the intervention/strategy such as planning, context analysis, decision making, research, monitoring, evaluation and/or learning to ensure inclusive representation, maximum participation, and uncompromised consultation
   e. Builds and sustains trust within the community

      To simplify the assessment of trust, the following criteria based on the work of Di Napoli et al.[20], have been adopted. At least two of the four criteria must be met to indicate trust with the community:

      i. Presence of interest and competence in offering services that support the community's needs and allows the realization of the community members' aspirations
      ii. Community members are willing to participate in the improvement of the community through their effort of contribution of valuable resources
      iii. Community members find pleasure and meaning in spending their time participating
      iv. Community members expect that the engagement will improve future resources related to security, decision-making, participation, and achieving their goals

Definitions of Terms	
Communities	Groups of people who may or may not be spatially connected, but share common interests, concerns, or identities. These communities could be local, national or international, with specific or broad interests[21]
Community engagement	"The process of working collaboratively with and through groups of people affiliated by geographic proximity, special interest, or similar situations to address issues affecting the well-being of those people"[22]<br>"The process of developing relationships that enable stakeholders to work together to address health-related issues and promote wellbeing to achieve positive health impact and outcomes"[23]

For peer review only - http://bmjopen.bmj.com/site/about/guidelines.xhtml

Social determinants of health	"Non-medical factors that influence health outcomes". They are circumstances where "people are born, grow, work, live, and age, and the wider set of forces and systems shaping the conditions of daily life"[24]
Trust	"Positive expectations of community members toward the current and future opportunities they perceive in their local community, namely the place where they live and interact"[20]<br>Building purposeful and compassionate relationships through a resilient and community-competent health workforce that adapts to the needs and preferences of the people they serve[25]

Writing Case Summaries

A summary was written for each identified case including the project's name, implementing institution, number of years the project was implemented, implementation site, and health issues/topic addressed. The rationale, objectives, intervention, outcomes, lessons, challenges, and factors promoting and/or impeding CE were abstracted. Social innovations, if any, were included.

Compilation of Materials

All selected and created documents were uploaded to the project's Google Drive and kept in storage, pending migration to a WHO repository for the database, CELP, and CEWP.

Analysis and Identification of Common Themes

Content analysis of the summaries and other data extracted from the screened materials was done using open coding. Key ideas and nuances were identified and grouped into categories and themes. These were then used to tag and organize the materials in the database.

For peer review only - http://bmjopen.bmj.com/site/about/guidelines.xhtml

Development of the Community Engagement Learning Package

The CELP is a curation of CE lessons and tools presented as online (asynchronous) modules designed to capacitate learners on basic concepts, principles, and applications of CE, and explore best practice experiences in solving health problems and promoting health through CE. In-depth analysis done with the contents of the database identified important CE principles, practices, lessons, challenges, and barriers encountered in different contexts and regions. Existing CE frameworks, toolkits, and guides were also surveyed. Emerging themes and concepts were utilized as the basis for the development of the CELP. SMEs contributed to the contents of the CELP designed to be delivered in an online learning management system.

Initial outline and plans for the CELP were also vetted among the CEP WG, and stakeholders and partners who have extensive experience in engaging and mobilizing communities, both at the regional and global levels. Comments, critiques, suggestions, and recommendations that emerged from the series of vetting processes further shaped and enhanced the content of the learning package.

Development of the Community Engagement Workshop Package

The CEWP was developed as a complementary strategy to the CELP, highlighting important topics and practical activities that might be useful for participants to enhance their CE practice. It was initially designed for face-to-face engagements, but because of the restrictions brought about by the pandemic, the pilot implementation was done online. The package materials were made into a downloadable format that can be adapted in either online or face-to-face settings. Different iterations of the activity design were developed based on the different possible country contexts, utilizing the input from

For peer review only - http://bmjopen.bmj.com/site/about/guidelines.xhtml

SIHI networks and frontline responders engaging specific issues and populations - migrants, indigenous populations, people living with disabilities, women, elderly and youths.

Testing the Learning and Workshop Packages

Prototypes of the packages were tested among stakeholders, particularly community mobilizers, public health practitioners and other potential end-users.

An online platform was created to test the online learning package. Pilot participants were selected using criteria that facilitated the inclusion of different groups and were invited to undergo the online asynchronous training. Feedback from the participants were obtained through online evaluation forms and were used to guide the revision of the training design.

Pilot testing for the workshop package was conducted in two phases through an online video conferencing platform. The first phase was implemented among participants from the Philippines. The pilot run tested the regional applicability and impact of the materials and content. The second phase was conducted among a global set of participants, which tested its universal applicability and impact. In both phases, user experiences were collected and used to refine the packages.

Ethical Considerations

The development of the CEP did not entail participation of human subjects that requires ethical approval by the WHO Ethics Review Committee.[26] The collection of feedback from pilot participants is a regular mechanism to evaluate training. Informed consent was obtained before documenting CE practitioners' experiences and recording workshop

For peer review only - http://bmjopen.bmj.com/site/about/guidelines.xhtml

proceedings. Information about the project and its objectives and the extent of their participation were discussed.

Monitoring & Evaluation in Project Development

Regular internal SIHI and WHO reviews and consultative processes were facilitated to ensure that project deliverables met the needs of the end-users and fulfilled the objectives of the project.

Limitations in Conducting the CEP Activities/Process

All engagements and coordination for this project were done remotely using online platforms due to the restrictions brought about by the COVID-19 pandemic. The team ensured that participatory approaches were reinforced and the voices of CE practitioners were incorporated in the CEP.

RESULTS

Community Engagement Database

A database of experiences on CE was developed across public health in different settings. WHO and partners identified relevant resources that captured CE experiences, using the prescribed inclusion criteria. Materials in various formats (documents, videos, etc.) that highlighted the practices, lessons and challenges in working with the communities were compiled. The documents and related materials are in English, Spanish, and French. Summaries of documented CE cases are available in English.

For peer review only - http://bmjopen.bmj.com/site/about/guidelines.xhtml

Categories of Cases in the CE Database

There are 356 cases in the database (290 identified from published literature, 57 from grey literature, and nine from CE practitioner interviews) from all six WHO regions, categorized according to the health topic (Table 3). In addition, a total of 56 cases dealing with health emergencies were identified with 30 cases on COVID-19, 12 on Ebola, nine on environmental risk and disaster, and five on humanitarian crises.

Table 3 ***Distribution of Cases According to Health Topic and the WHO Regions***

Health Topic Category	No. of Cases per WHO Region						
	AFR	EMR	EUR	PAHO	SEAR	WPR	Total
Communicable Diseases	66	10	2	20	14	21	133
Primary Health Care	9	2	11	13	6	8	49
Maternal & Child Health	9	1	2	5	5	3	25
WASH	6	0	1	3	1	0	11
Sexual & Reproductive Health	3	2	2	4	1	2	14
Social Determinant of Health	1	5	13	27	7	3	56
Mental Health	0	3	1	5	1	4	14
NCDs	1	3	4	3	8	11	30
Nutrition	0	0	0	2	2	2	6
Others	3	0	5	3	5	2	18
**Total**	**98**	**26**	**41**	**85**	**50**	**56**	**356**

For peer review only - http://bmjopen.bmj.com/site/about/guidelines.xhtml

CE Practitioner Interviews

Seven CE practitioner interviews were conducted – five interviewees from AFRO, one each from PAHO and WPRO. These interviews identified nine unpublished CE experiences and explored CE strategies and dynamics and how that influenced the sustainability of health interventions.

Thematic Analysis

The case summaries were coded and analyzed, capturing themes from the rationale for CE, key insights, facilitating factors, and barriers. The documentation of the thematic analysis is available in an additional document in the database. Table 4 presents the thematic areas that emerged from the review of the cases.

Table 4 ***Summary of Themes from the Community Engagement Cases***

Rationale for Community Engagement	Contextual and health system challenges<br>Health and social goals<br>Mechanisms
Key Points and Insights	Community mobilization<br>Individual and community agency<br>Multi-stakeholder engagement<br>Multidirectional communication<br>Building on local capacity<br>Access, acceptability and adaptation<br>Inclusion<br>Sustainability<br>Participatory research<br>Basic principles
Facilitators of Community Engagement	Adapting the intervention<br>Applying participatory principles and approaches<br>Maximizing reach and access<br>Utilizing support mechanisms

For peer review only - http://bmjopen.bmj.com/site/about/guidelines.xhtml

Barriers to Community Engagement	Societal and contextual issues<br>Challenges with leadership<br>Weak health system<br>Challenges in encouraging and sustaining participation<br>Inadequate reach and access<br>Knowledge/information gaps<br>Lack of trust<br>Issues in communication<br>Inadequate or improper allocation of resources<br>Organizational and logistic problems<br>Challenges on the sustainability and generalizability of the project<br>Timing and duration of community engagement

Community Engagement Learning Package (CELP)

From the CE materials collected, the CELP was developed anchored on basic principles and standards of CE and grounded on actual experiences in working with communities in different contexts and settings. The CELP includes four self-instructional modules that participants may complete independently or as a ladder-type course. Each module presents basic frameworks and concepts of CE in relation to the theme of that module and are then tied to real world examples of CE in different contexts (see Table 5). Target learners include early to mid-level professionals and practitioners applying community engagement in their work who may come from various disciplines such as medical and health sciences, public health, public policy and administration, program management, social development and other social sciences. Students both at the undergraduate and postgraduate levels of any higher education institution, from various disciplines as mentioned above may also benefit from the modules.

For peer review only - http://bmjopen.bmj.com/site/about/guidelines.xhtml

Table 5 ***Modules of the Community Engagement Learning Package***

Module Title	Main framework/s used	Sample cases used
Module 1: Engaging and Mobilizing Communities for Health and Development	WHO community engagement framework for quality, people-centred and resilient health services[23]<br><br>Community engagement: a health promotion guide for universal health coverage in the hands of the people[27]	Setting health priorities in a community: a case example<br>Sousa et al., 2017[32]<br><br>Participatory learning and action to address type 2 diabetes in rural Bangladesh: a qualitative process evaluation<br>Morrison et al., 2019[33]<br><br>Community engagement in outbreak response: lessons from the 2014–2016 Ebola outbreak in Sierra Leone<br>Bedson et al., 2020[34]<br><br>'What works here doesn't work there': The significance of local context for a sustainable and replicable asset-based community intervention aimed at promoting social interaction in later life.<br>Wildman et al., 2019[35]
Module 2: Strengthening Health Systems through Community Engagement	Systems thinking for health systems strengthening[28]	Achieving UHC in Samoa through Revitalizing PHC and Reinvigorating the Role of Village Women Groups<br>Baghirov et al., 2019[36]
Module 3: Community Engagement in All-Hazards Emergency and Disaster Risk Management	Sendai framework for disaster risk reduction 2015–2030[29]<br><br>Health Emergency and Disaster Risk Management Framework[30]	Shifting Paradigms: Strengthening Institutions for Community-Based Disaster Risk Reduction and Management<br>Bawagan et al., 2015[37]

For peer review only

For peer review only - http://bmjopen.bmj.com/site/about/guidelines.xhtml

Module 4: Community Engagement as a Driver for Achieving Health Equity and Community Resilience	Minimum Quality Standards and Indicators for Community Engagement[31]	Integrated vector control of Chagas disease in Guatemala: a case of social innovation in health<br>Castro-Arroyave et al., 2020[38]

The pilot participants found the CELP to be comprehensive in terms of content and with a user-friendly format. They appreciated how other concepts in public health were linked to CE. They suggested more practical applications and specific how-to's, and assessment activities with immediate feedback. These were all taken into consideration in the revision of the modules.

Community Engagement Workshop Package (CEWP)

The CEWP provides tools and templates for identifying other CE experiences in a workshop format. The contents are similar to the CELP, with a special focus on documenting "new" CE experiences and a walk-through of using and submitting case studies for the CE database. The target participants are practitioners who are interested in sharing their CE experiences. The CEWP allows the continuous collection of evidence and discussions with stakeholders on CE principles, practices, and frameworks. These resources will be cataloged, categorized, and used to update the database and the learning and workshop packages.

Participants and observers of the CEWP pilot were satisfied with the introduction and ice-breaking activities which set the stage for conducive training sessions. Participants also expressed satisfaction on the content, pointing out that the workshop addressed aspects of CE not previously considered. The topics of the training were noted to be far-reaching,

For peer review only - http://bmjopen.bmj.com/site/about/guidelines.xhtml

covering several CE frameworks, with good video presentations. Participants were able to relate the lessons and case studies to their experiences. They pointed out a few areas of improvement, including the need for adequate time to study the cases prior to the synchronous online sessions and more breakout sessions for participants to raise issues and ensure more diverse voices and opinions. They also recommended that the frameworks need to further emphasize listening and understanding community perspectives right from the start of the engagement.

DISCUSSION

The CEP and its development showcase innovative elements in the project design, the human resources involved and way of working, and the interrelationships of the different CEP components.

The CEP conceptualization and design involved broad consultations and co-creation with a community of diverse teams of WHO, SIHI hubs, UNICEF, and other implementing partners, and frontline responders. The process and products of the package were vetted among stakeholders and partners at the regional and global levels. In addition, community practitioners were consulted regarding the screening criteria of cases to be included in the database, shared undocumented CE practices, and participated in the pilots of the learning and workshop packages to provide user feedback. This multi-stakeholder consultative processes allowed for the creation of a grounded, contextualized, relevant and integrated package.

Working on the CEP project during the COVID-19 pandemic did not deter the WHO and SIHI from intensifying collaboration. The use of online platforms enabled the team to engage and mobilize relevant resources and develop the CEP components despite the

For peer review only - http://bmjopen.bmj.com/site/about/guidelines.xhtml

absence of face-to-face consultations and other limitations. Creative use of online platforms was also maximized for the different components of the CEP (e.g., online database, online modules) while still providing templates for possible face-to-face delivery, allowing for flexibility in engagement methods.

The three components of the CEP feed into each other. The thematic analysis of the materials in the CE database guided the design of the CELP and CEWP. Selected cases were also used to reinforce and provide real-world application to the CE frameworks and related concepts in the online modules. The CEWP facilitates the discussion of CE principles and practices among practitioners and the collection of new information for updating the database and CELP with "new" CE experiences.

The merit of the current CEP project over existing documentation is that the CEP is broad-based - not limited to health emergencies, but includes other public health and social developmental activities such as routine immunization, neglected tropical diseases, city and urban development, nutritional interventions and disaster risk management, among others.

An operational challenge during the documentation was the language barrier. The cases were limited to English, French and Spanish. Future researchers can explore relevant documented and undocumented experiences in other languages, which will make the database more comprehensive and unifying at the same time.

CONCLUSION

The design of the CEP emphasized interrelationships among its components – CE database, learning package and workshop package. The CELP contents were taken from the comprehensive thematic analysis of the database. The CEWP facilitates the

For peer review only - http://bmjopen.bmj.com/site/about/guidelines.xhtml

documentation of "new" CE experiences and their nuances, ensuring timely updates of the database by CE practitioners themselves.

Most of the cases included in the CEP database presented key insights on CE including its basic principles and the role of individual and community agency, building on local capacity, multi-directional communication, inclusion, and multi-stakeholder engagement. Barriers to CE including issues of access, acceptability and adoption in the setting of weak health systems and societal issues were also identified. The learning and workshop packages were then developed to guide health professionals and other stakeholders based on these grounds.

The development of the CEP was the work of multiple global stakeholders providing synergistic contributions and bridging silos. The description of the CEP methodology will allow replication, provide transparency into the development of the CEP and present lessons learned during the development of a robust and harmonized package.

ACKNOWLEDGEMENTS

The authors would like to thank Dr. Nedret Emiroglu, **Director, WHO Country Readiness Strengthening Department, WHO**; the **WHO CEP Working Group members and resource persons**: Qudsia Huda, Ankur Rakesh, Sohel Saikat, Saqif Mustafa, Samar Elfeky, Mervat Gawrgyous, Suvajee Good, Alex Camacho, Julienne Ngoundoung Anoko, Aphaluck Bhatiasevi, Faten Ben Abdelaziz, Ana Gerlin Hernandez Bonilla, Mihai Mihut, Nicole Valentine, Mary Manandhar, Dayo Spencer-Walters, Philippe Eric Gasquet, Joao Jose Salavessa Rangel De Almeida, Nina Gobat, Redda Seifeldin, Sameera Suri, Renee Christensen, Melinda Frost, Simon Van Woerden, Cristiana Salvi, Leonardo Palumbo, Aminata Grace Kobie, Anna Coates, Sonja Caffe, Gerry Eijkemans, Orielle Solar

For peer review only - http://bmjopen.bmj.com/site/about/guidelines.xhtml

Hormazabal, Tonia Rifaey, Peggy Edmond Hanna, Godfrey Yikii, Dalia Samhouri, Supriya Bezbaruah, Kira Fortune and UNICEF resource persons Rania Elessawi, Naureen Naqvi, Ana Puri; the **RCCE Collectives Services colleagues** from the International Federation of the Red Cross and Red Crescent Societies (IFRC), UNICEF, Global Outbreak Alert and Response Network (GOARN) and WHO; and the **CEP project staff, consultants and research associates:** Erlyn Sana, Nina Castillo-Carandang, Gladys Armada, Jennel Pimentel, Justin Bryan Maranan, Nathalia Palma, Philippe Galban, Clarence Diaz, Eric David Ornos, Celina Gonzales, Samuel Tristan Vinluan, Ma. Pamela Tagle, Linda Mipando-Nyondo, Deborah Nyirenda, Katusha de Villiers, José Alejandro Carias, Perla Simons Morales, Sandra Barahona, Karla Zúniga, and Milena Bautista.

CONTRIBUTORSHIP

YB, MDPL, JRBC, JDMA, UEO, AOJ, and NRJ conceptualized and designed this work. JRBC, JDMA, PMPT, ARU, MF, MIE, JA, DM, BKM, OIE, ON, LA, NG, CNA, BB, EC, EN, VNK, and GMK gathered and analyzed data. YB, UEO, SAO, JRBC, JDMA, and PMPT drafted the manuscript. All authors reviewed, edited, and approved the final version of the manuscript.

COMPETING INTERESTS

There are no competing interests for any author.

FUNDING

The development of the WHO Community Engagement Package was funded by the World Health Organization. The Social Innovation in Health Initiative (SIHI) is funded by TDR, the Special Programme for Research and Training in Tropical Diseases co-sponsored by UNICEF, UNDP, the World Bank, and WHO. TDR receives additional funding from the Swedish International Development Cooperation Agency (Sida), to support SIHI.

Grant/Award Number: N/A

For peer review only - http://bmjopen.bmj.com/site/about/guidelines.xhtml

DATA SHARING/DATA AVAILABILITY

Data are available upon reasonable request.

ETHICS APPROVAL STATEMENT

This study does not involve human participants.

REFERENCES

1. WHO. The impact of the COVID-19 pandemic on noncommunicable disease resources and services: results of a rapid assessment [Internet]. 2020 Sep [cited 2021 Oct 19] p. 22. Available from: https://www.who.int/publications-detail-redirect/9789240010291

2. Zawawi A, Alghanmi M, Alsaady I, Gattan H, Zakai H, Couper K. The impact of COVID-19 pandemic on malaria elimination. Parasite Epidemiol Control. 2020;11(November):e00187.

3. Ters LET. COVID-19 policies : Remember measles Restart science stronger after COVID-19 Uphold the nuclear weapons test moratorium. 2020;369(6501):261–3.

4. Sun X, Samba TT, Yao J, Yin W, Xiao L, Liu F, et al. impact of the Ebola outbreak on routine immunization in western area, Sierra Leone - A field survey from an Ebola epidemic area. BMC Public Health. 2017;17(1):1–6.

5. WHO. COVID-19 Global Risk Communication and Community Engagement Strategy – interim guidance [Internet]. 2020 [cited 2021 Oct 19]. Available from: https://www.who.int/publications-detail-redirect/covid-19-global-risk-communication-and-community-engagement-strategy

6. IFRC. From words to action: Towards a community-centred approach to preparedness and response in health emergencies [Internet]. 2019 [cited 2021 Oct 19]. Available from: https://www.gpmb.org/annual-reports/overview/item/from-words-to-action-towards-a-community-centred-approach-to-preparedness-and-response-in-health-emergencies

7. Gillespie AM, Obregon R, Asawi RE, Richey C, Manoncourt E, Joshi K, et al. Social Mobilization and Community Engagement Central to the Ebola Response in West Africa: Lessons for Future Public Health Emergencies. Glob Health Sci Pract. 2016 Dec 23;4(4):626–46.

8. Wright K, Parker M. In emergencies, health research must go beyond public engagement toward a true partnership with those affected. Nat Med. 2020 Mar;26(3):308–9.

For peer review only - http://bmjopen.bmj.com/site/about/guidelines.xhtml

9. O'Mara-Eves A, Brunton G, Oliver S, Kavanagh J, Jamal F, Thomas J. The effectiveness of community engagement in public health interventions for disadvantaged groups: a meta-analysis. BMC Public Health. 2015 Feb 12;15(1):129.

10. UN OCHA. Workshop Report Final_Community Engagement in Humanitarian Action.pdf [Internet]. ReliefWeb. 2016 [cited 2021 Oct 19]. Available from: https://reliefweb.int/sites/reliefweb.int/files/resources/Workshop%20Report%20Final_Community%20Engagement%20in%20Humanitarian%20Action.pdf

11. IFRC. How your community can prevent the spread of COVID-19. 2020;19(March):1–5.

12. Bedford J, Chitnis K, Webber N, Dixon P, Limwame K, Elessawi R, et al. Community Engagement in Liberia: Routine Immunization Post-Ebola. J Health Commun. 2017;22(0):81–90.

13. Habib MA, Soofi S, Cousens S, Anwar S, Haque N ul, Ahmed I, et al. Community engagement and integrated health and polio immunisation campaigns in conflict-affected areas of Pakistan: a cluster randomised controlled trial. Lancet Glob Health. 2017;5(6):e593–603.

14. Mayhew SH, Kyamusugulwa PM, Bindu KK, Richards P, Kiyungu C, Balabanova D. Responding to the 2018 – 2020 Ebola Virus Outbreak in the Democratic Republic of the Congo : Rethinking Humanitarian Approaches. 2021;1731–47.

15. Oleribe OO, Salako BL, Ka MM, Akpalu A, McConnochie M, Foster M, et al. Ebola virus disease epidemic in West Africa: Lessons learned and issues arising from West African countries. Clin Med J R Coll Physicians Lond. 2015;15(1):54–7.

16. Mason C, Barraket J, Friel S, O'Rourke K, Stenta CP. Social innovation for the promotion of health equity. Health Promot Int. 2015;30:ii116–25.

17. Halpaap BM, Tucker JD, Mathanga D, Juban N, Awor P, Saravia NG, et al. Social innovation in global health: sparking location action. Lancet Glob Health. 2020;8(5):e633–4.

18. DiClemente R, Nowara A, Shelton R, Wingood G. Need for innovation in public health research. Am J Public Health. 2019;109:S117–20.

19. Omoleke SA, Bayugo YV, Oyene UE, Abrahams J, Gobat N, Good S, et al. WHO Community Engagement Package: A Reinforcement of an Inclusive Approach to Global Public Health. Int J Epidemiol Health Sci [Internet]. 2021 Jul 1 [cited 2021 Oct 12];2(7). Available from: http://www.ijehs.com/article_244835.html

20. Di Napoli I, Dolce P, Arcidiacono C. Community Trust: A Social Indicator Related to Community Engagement. Soc Indic Res. 2019 Sep 1;145(2):551–79.

For peer review only - http://bmjopen.bmj.com/site/about/guidelines.xhtml

21. WHO. The 7th Global Conference on Health Promotion [Internet]. 2009 [cited 2021 Oct 12]. Available from: https://www.who.int/teams/health-promotion/enhanced-wellbeing/seventh-global-conference/community-empowerment

22. DHHS. Principles of Community Engagement [Internet]. 2nd ed. NIH; 2011. Available from: http://www.atsdr.cdc.gov/communityengagement/pdf/PCE_Report_508_FINAL.pdf

23. WHO. WHO community engagement framework for quality, people-centred and resilient health services. 2017. Available from: https://apps.who.int/iris/handle/10665/259280. License: CC BY-NC-SA 3.0 IGO

24. WHO. Health in the post-2015 development agenda : need for a social determinants of health approach Joint statement of the UN Platform on Social Determinants of Health. Jt Statement UN Platf Soc Determinants Health. 2015;1–18.

25. WHO. Enhanced Capacity Building Training for Frontline Staff on Building Trust and Communication. 2015;(July).

26. WHO. Ethics Review Committee Frequently Asked Questions [Internet]. 2019 [cited 2022 Jan 10]. Available from: https://extranet.who.int/ercweb/faq.php#fq6

27. WHO. Community engagement: a health promotion guide for universal health coverage in the hands of the people. 2020. Available from: https://apps.who.int/iris/handle/10665/334379. License: CC BY-NC-SA 3.0 IGO

28. Don DS and Taghreed A. Systems thinking for health systems strengthening. Alliance for Health Policy and Systems Research, WHO. 2009. Available from: https://apps.who.int/iris/bitstream/handle/10665/44204/9789241563895_eng.pdf?sequence=1

29. United Nations. Sendai framework for disaster risk reduction 2015–2030. 2015. Available from: https://www.preventionweb.net/files/43291_sendaiframeworkfordrren.pdf

30. WHO. Health Emergency and Disaster Risk Management Framework. 2019. Available from: https://www.who.int/hac/techguidance/preparedness/health-emergency-and-disaster-risk-management-framework-eng.pdf Licence: CC BY-NC-SA 3.0 IGO.

31. UNICEF. Minimum quality standards and indicators in community engagement [Internet]. 2020 [cited 2021 Oct 19]. Available from: https://www.unicef.org/mena/reports/community-engagement-standards

32. Sousa FAMR, Goulart MJG, Braga AMS, Medeiros CMO, Rego DCM, Vieira FG, et al. Setting health priorities in a community: a case example. Rev Saude Publica. 2017; 51:11. Available from: https://www.ncbi.nlm.nih.gov/pmc/articles/PMC5336317/pdf/0034-8910-rsp-S1518-87872017051006460.pdf

For peer review only - http://bmjopen.bmj.com/site/about/guidelines.xhtml

33. Morrison J, Akter K, Jennings HM, et al. Participatory learning and action to address type 2 diabetes in rural Bangladesh: a qualitative process evaluation. BMC Endocr Disord. 2019; 19,118 https://doi.org/10.1186/s12902-019-0447-3

34. Bedson J, Jalloh MF, Pedi D, et al. Community engagement in outbreak response: lessons from the 2014–2016 Ebola outbreak in Sierra Leone. BMJ Global Health. 2020; 5, Article e002145. doi:10.1136/ bmjgh-2019-002145

35. Wildman JM, Valtorta N, Moffatt S & Hanratty B. 'What works here doesn't work there': The significance of local context for a sustainable and replicable asset-based community intervention aimed at promoting social interaction in later life. Health Soc Care Community. 2019; 27, 1102– 1110. https://doi.org/10.1111/hsc.12735

36. Baghirov R, Ah-Ching J & Bollars C. Achieving UHC in Samoa through Revitalizing PHC and Reinvigorating the Role of Village Women Groups. Health Systems & Reform. 2019; 5:1, 78-82, DOI: 10.1080/23288604.2018.1539062

37. Bawagan AB, Polotan-dela Cruz L, Felizco MS, Tan MJ, Wamil M & Germar JS. Shifting Paradigms: Strengthening Institutions for Community-Based Disaster Risk Reduction and Management. Quezon City: UP College of Social Work and Community Development. 2015. Available from: http://iskwiki.upd.edu.ph/flipbook/viewer/?fb=2012-79319-U.P.-SHI&fbclid=IwAR1CKfUX_3RcdAoJiq9-4N0f6o7zCvvANoy7EnrAoMKn-rKik9EyDM4aXas#page-1

38. Castro-Arroyave D, Monroy MC & Irurita MI. Integrated vector control of Chagas disease in Guatemala: a case of social innovation in health. Infect Dis Poverty. 2020; 9:25. https://doi.org/10.1186/s40249-020-00639-w

For peer review only

For peer review only - http://bmjopen.bmj.com/site/about/guidelines.xhtml

Figure Legend

Figure 1. WHO Community Engagement Package Components and Relationships

For peer review only

For peer review only - http://bmjopen.bmj.com/site/about/guidelines.xhtml

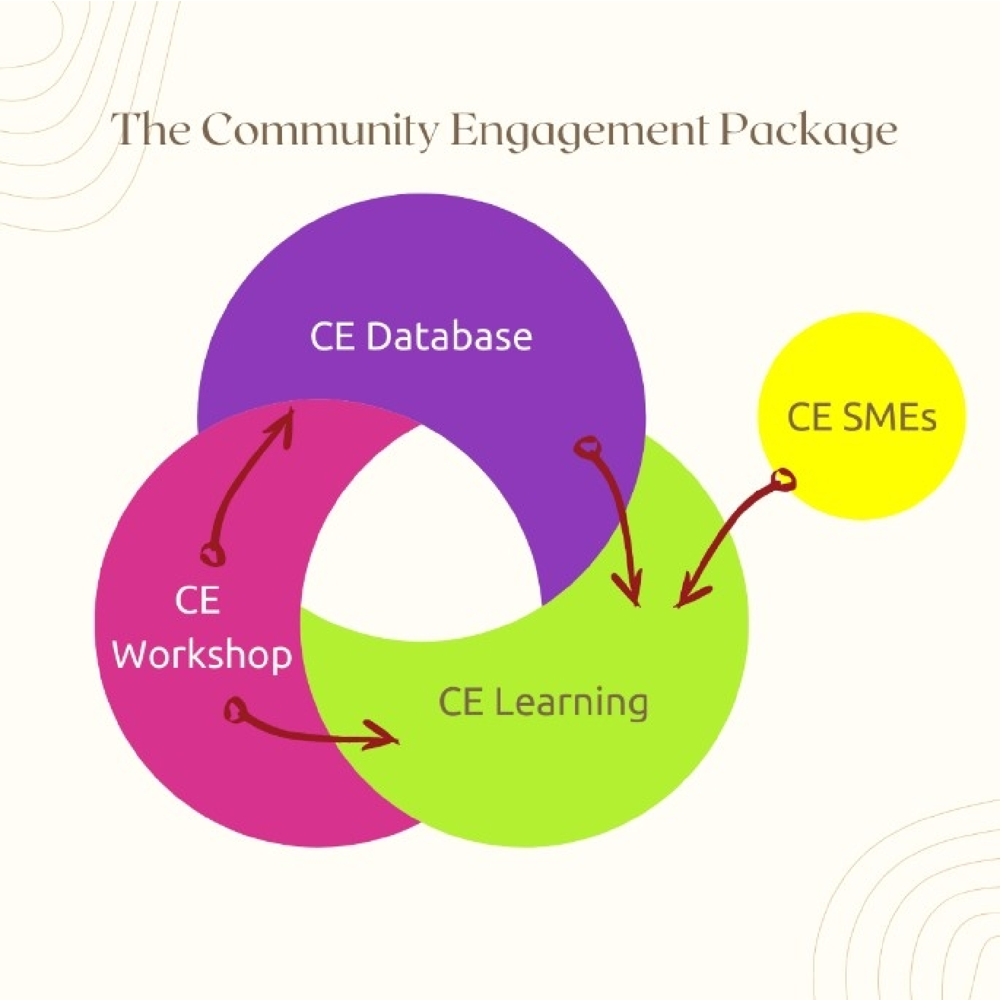

181x180mm (96 x 96 DPI)
